# Late acquisition of *BCR::ABL1* during clonal evolution of *SAMD9-*associated MDS with phenotypic shift from AML to B-ALL

**DOI:** 10.1007/s12185-026-04248-5

**Published:** 2026-07-30

**Authors:** Yusuke Nakata, Anna Hiratsuka, Takeshi Yoroidaka, Wakana Takahashi, Itaru Nishikawa, Masataka Sakashita, Shinya Yamada, Hiroki Mizumaki, Akiyo Yoshida, Tatsuya Imi, Hiroyuki Maruyama, Hiroyuki Takamatsu, Kohei Hosokawa, Yasuhito Nannya, Seishi Ogawa, Toshihiro Miyamoto

**Affiliations:** 1 Department of Hematology, Faculty of Medicine, Institute of Medical Pharmaceutical and Health Sciences, Kanazawa University, 13-1 Takara-Machi, Kanazawa, Ishikawa 920-8641 Japan; 2 Division of Hematology, Keiju Kanazawa Hospital, Kanazawa, Japan; 3 Division of Hematopoietic Disease Control, Institute of Medical Science, The University of Tokyo, Tokyo, Japan; 4 Pathology and Tumor Biology, Graduate School of Medicine, Kyoto University, Kyoto, Japan

**Keywords:** *BCR::ABL1*, Phenotypic shift, AML, ALL, MDS

## Abstract

We describe a unique case of *SAMD9*-associated myelodysplastic syndrome (MDS) with monosomy 7 that evolved over 16 years into *BCR::ABL1*-positive acute myeloid leukemia (AML) and subsequently manifested as B-cell acute lymphoblastic leukemia (B-ALL). Genomic analysis at AML diagnosis revealed a germline *SAMD9* mutation together with somatic *RUNX1* and *PPM1D* mutations, supporting stepwise clonal evolution, with *BCR::ABL1* emerging as a late leukemogenic event. The dominant leukemic population at AML onset showed myeloid morphology and immunophenotype, whereas a minor CD19^+^CD10^+^ population was already detectable. Following venetoclax and azacitidine therapy, the dominant leukemic phenotype shifted to B-ALL while retaining *BCR::ABL1* positivity. Detection of the Philadelphia chromosome in mature neutrophils at both AML onset and ALL relapse supported multilineage involvement of a multipotent *BCR::ABL1*-positive clone. Ponatinib achieved disease control. This case highlights late acquisition of *BCR::ABL1* during SAMD9-associated clonal evolution and therapy-driven phenotypic shift within a shared Ph-positive leukemic stem-cell hierarchy.

## Introduction

Acute myeloid leukemia (AML) develops through stepwise clonal evolution originating from hematopoietic stem cells (HSCs), driven by the sequential acquisition of genetic alterations with distinct biological functions. Early mutations commonly occur in genes associated with clonal hematopoiesis (CH), followed by progression through myelodysplastic syndrome (MDS) and acquisition of proliferative driver lesions, ultimately leading to leukemic transformation [1, 2].

*BCR::ABL1* fusion gene generated by t(9;22)(q34;q11), known as Philadelphia chromosome, is a canonical oncogenic driver of chronic myeloid leukemia (CML) and is also a well-established lesion in B-lineage acute lymphoblastic leukemia (B-ALL). It is typically acquired at the level of multipotent HSCs and drives leukemogenesis across multiple hematopoietic lineages in CML and in a subset of B-ALL with multilineage involvement [3]. In contrast, acquisition of *BCR::ABL1* as a late event during clonal evolution from MDS to AML is exceedingly rare [4].

Lineage switch or phenotypic shift between myeloid and lymphoid leukemias is another uncommon but increasingly recognized phenomenon, often associated with intrinsic stem-cell plasticity or therapeutic pressure [5]. Especially, with increasing use of antigen-targeted immunotherapies in B-ALL, including CD19-directed bispecific antibodies and chimeric antigen receptor T-cell therapy, emergence of transformation from B-ALL to AML has been reported associated with dismal outcomes [6, 7]. However, lineage switch from *BCR::ABL1*-positive AML to B-ALL has only rarely been described [8].

Here, we describe a rare case of long-standing *SAMD9*-associated MDS with monosomy 7 that evolved over 16 years into *BCR::ABL1*-positive AML and subsequently underwent phenotypic dominance shift to *BCR::ABL1*-positive B-ALL following venetoclax and azacitidine therapy, illustrating both late acquisition of *BCR::ABL1* during clonal evolution and therapy-associated lineage plasticity of a shared Philadelphia-positive multipotent stem-cell clone.

## Case report

A 56-year-old man was referred for evaluation of leukocytopenia (white blood cell (WBC) count 2100/µL). Bone marrow examination revealed dysplastic features without increased blasts (0.2%). Cytogenetic analysis revealed monosomy 7. *BCR::ABL1* transcripts were not detected at diagnosis. Based on these findings, he was diagnosed with refractory cytopenia with multilineage dysplasia. The patient was followed with observation alone, without clinical features suggestive of chronic-phase CML.

**Fig. 1:**
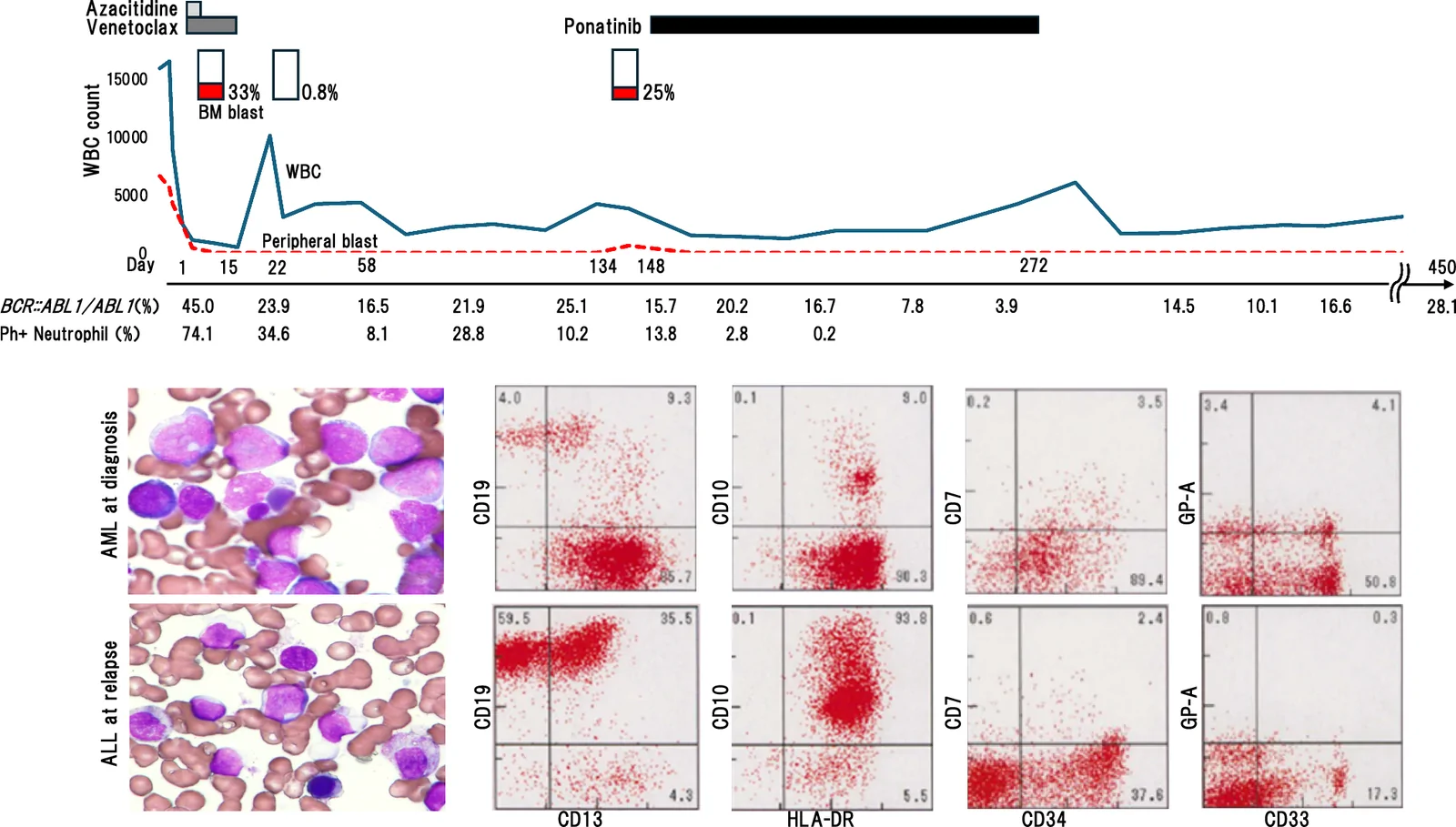
Clinical course and immunophenotypic shift during disease evolution. Longitudinal analysis of bone marrow blast percentage, *BCR::ABL1* transcript levels, and proportion of Ph-positive neutrophils. Representative morphology and flow cytometry findings at AML diagnosis and B-ALL relapse are shown. This analysis illustrates the temporal relationship between clonal evolution, therapeutic intervention, and phenotypic shift during disease progression

Sixteen years later, at age 72, peripheral blood blasts were detected. Laboratory findings showed WBC 15,820/µL (blasts 33%, promonocytes 19%), hemoglobin 13.4 g/dL, and platelets 20 × 10^4^/µL. Bone marrow examination demonstrated 33.2% blasts and 24.8% promonocytes positive for myeloperoxidase staining, consistent with AML. The dominant leukemic population showed myeloid immunophenotype with CD13, CD33, CD34, CD38, and HLA-DR, whereas a minor population expressing CD19 and CD10 cells was detectable (Figure 1). Cytogenetic analysis showed 46, XY, t(9;22)(q34.1;q11.2), add(3)(q21), add(7)(q22) in all metaphases. Quantitative PCR confirmed minor *BCR::ABL1* (*BCR::ABL1/ABL1* ratio 45.03%). Fluorescence in situ hybridization detected the Philadelphia chromosome in 74.1% of neutrophils. Next-generation sequencing (NGS) identified a germline *SAMD9* p.Q154X (VAF 0.494) mutation and somatic mutations in *RUNX1* p.R166X (VAF 0.417) and *PPM1D* p.Q571X (VAF 0.417). Based on these results, the patient was diagnosed with *BCR::ABL1*-positive AML according to the International Consensus Classification (ICC) criteria [3], although the possibility of mixed phenotype acute leukemia (MPAL) with *BCR::ABL1* fusion (B/myeloid bilineage) could also be considered from the perspective of the WHO Classification (5th edition) [9]. Thus, induction therapy with venetoclax (400 mg/day) and azacitidine (75 mg/sqm for 7 days) targeting AML was selected as initial treatment. Venetoclax was discontinued on day 15 due to pneumonia. Bone marrow examination on day 22 showed a marked reduction of blasts (0.8%), consistent with morphologic leukemia-free state (Figure 1). *BCR::ABL1/ABL1* ratio decreased to 23.93% but remained detectable. Further therapy was not pursued due to comorbidities.

On day 134, subsequent relapse occurred with lymphoid morphology (blasts 25%) and expression of CD10 and CD19, consistent with a phenotypic dominance shift to B-ALL (Figure 1). Cytogenetic analysis again demonstrated t(9;22) in all metaphases, and minor *BCR::ABL1* remained detectable throughout disease course. The disease was therefore interpreted as overt B-ALL with lymphoid predominance retaining *BCR::ABL1* positivity [3]. NGS analysis was not performed at relapse due to limited sample availability. Ponatinib therapy (30 mg/day) achieved sustained disease control, with gradual reduction of *BCR::ABL1* level. Ponatinib was discontinued at the patient’s request after 124 days, on day 272. At the most recent assessment on day 450, *BCR::ABL1/ABL1* ratio was 28.1%, and peripheral blood findings remained within normal limits without further therapy.

## Discussion

This case illustrates a distinctive evolutionary trajectory in which long-standing *SAMD9*-associated MDS progressed to *BCR::ABL1*-positive AML after a prolonged latency, followed by phenotypic dominance shift to B-ALL under therapeutic pressure.

Genomic findings suggest a multistep clonal evolution model. At the initial diagnosis of MDS, monosomy 7 was detected without *BCR::ABL1* rearrangement. At AML transformation, monosomy 7 was no longer detectable, whereas t(9;22)(q34;q11) newly emerged together with additional chromosomal abnormalities, including add(3)(q21) and add(7)(q22). In addition, NGS at AML diagnosis identified a germline *SAMD9* mutation together with somatic mutations *in RUNX1* and *PPM1D*. *SAMD9* is located on chromosome 7q21, and pathogenic variants in *SAMD9* have been implicated in the development of monosomy 7 through selective chromosomal loss that mitigates *SAMD9*-mediated growth suppression [9]. It is therefore plausible that *SAMD9*-mutated clone drove the emergence of monosomy 7 clone early in the disease course. Subsequent acquisition of additional mutations, including *RUNX1* and *PPM1D,* likely promoted clonal expansion and leukemic transformation, eventually followed by acquisition of *BCR::ABL1* as a late leukemogenic event [9, 10]. At B-ALL relapse, t(9;22) remained detectable whereas add(3)(q21) and add(7)(q22) disappeared. These findings suggest clonal evolution with replacement of the original monosomy 7-associated clone by a newly expanded *BCR::ABL1*-positive leukemic clone. The present case supports a model in which a predisposed clone undergoes stepwise evolution, acquiring additional mutations before leukemic transformation. Importantly, the late emergence of *BCR::ABL1* suggests that even canonical leukemia drivers may arise as secondary events during disease progression.

Another notable feature of this case is the phenotypic shift from a *BCR::ABL1*-positive myeloid-dominant leukemia at diagnosis to overt *BCR::ABL1*-positive B-ALL at relapse. Within *BCR::ABL1*-positive ALL, distinctive entities are recognized as “lymphoid-only,” in which *BCR::ABL1* is restricted to lymphoid progenitors, and “multilineage involvement,” in which *BCR::ABL1* is present in HSC and multiple lineages [3]. In the present case, detection of the Philadelphia chromosome in mature neutrophils at both AML onset and ALL relapse indicates that the *BCR::ABL1*-positive clone originated from multipotent HSCs, retaining the capacity for both myeloid and lymphoid differentiation.

Several mechanisms have been proposed, including the coexistence of multiple leukemic clones, intrinsic lineage plasticity of multipotent progenitors, or transcriptional reprogramming of leukemic blasts [5–7]. In this case, retrospective review of flow cytometry demonstrated that a minor CD19^+^CD10^+^ population was already detectable at AML onset, suggesting that myeloid- and lymphoid-competent leukemic populations coexisted within a shared *BCR::ABL1*-positive stem-cell hierarchy. The subsequent emergence of overt B-ALL may therefore reflect selective expansion of a preexisting lymphoid-competent subclone under therapeutic pressure, rather than a complete de novo lineage conversion. Venetoclax and azacitidine are highly active against proliferative myeloid clones and may have suppressed the dominant myeloid leukemia population while sparing or selecting for a minor lymphoid-competent *BCR::ABL1*-positive subclone. Subsequent response to ponatinib further supports the central role of *BCR::ABL1* in disease biology.

To our knowledge, this case provides rare clinical evidence that *BCR::ABL1* can be acquired as a late leukemogenic event within a *SAMD9*-driven clonal hierarchy. Furthermore, it highlights the capacity of a Ph-positive multipotent stem-cell-derived leukemic clone to undergo phenotypic dominance shift. The present case may therefore represent therapy-driven clonal selection and phenotypic plasticity within a shared multipotent *BCR::ABL1*-positive leukemic stem-cell hierarchy.

## Data Availability

The data supporting the findings of this study are available from the corresponding author upon reasonable request. Data are not publicly available because they contain information that could compromise the privacy of the patient.

## Abbreviations

AML: Acute myeloid leukemia
MDS: Myelodysplastic syndrome
CML: Chronic myeloid leukemia
ALL: Acute lymphoblastic leukemia
